# “They didn’t give up on me”: a women’s transitions clinic from the perspective of re-entering women

**DOI:** 10.1186/s13722-019-0142-8

**Published:** 2019-04-02

**Authors:** Katherine Thomas, John L. Wilson, Precious Bedell, Diane S. Morse

**Affiliations:** 1 Department of Psychiatry, University of Rochester School of Medicine, Rochester, USA; 2 Department of Medicine, University of Rochester School of Medicine, Rochester, USA

**Keywords:** Justice-involved women, Substance use disorders, Re-entry, Community health workers, Self-determination theory

## Abstract

**Background:**

Women recently released from incarceration have increased rates of co-occurring substance use, physical health, and mental health disorders. During re-entry, they face challenges navigating needed health services and social services stemming from these problems. Women’s Initiative Supporting Health Transitions Clinic (WISH-TC) is a primary care program that facilitates treatment access for re-entering women. Strategies include support and navigation assistance from peer community health workers.

**Methods:**

Thirteen participants, of whom 11 had a substance use disorder, completed semi-structured interviews about their experiences in WISH-TC as part of a process evaluation. We conducted a qualitative framework analysis informed by self-determination theory.

**Results:**

WISH-TC supported autonomy as staff helped motivate women to work toward personal health goals. Women were empowered to have their health needs met, and consequently, prioritized attending clinic. Regarding competence, WISH-TC built upon women’s existing knowledge to increase their health literacy and better understand their individual health needs. Relatedness support, both prior to re-entry and ongoing with clinic staff, was key in women’s satisfaction with their care. The clinic made procedural changes in response to the interviews, including providing orientation for the patients and training the clinic in trauma-informed practices.

**Conclusions:**

Our findings highlight the potential of a program for re-entering women, including those with substance use disorders to strengthen their abilities to navigate complex healthcare and societal systems. WISH-TC helped women feel supported, motivated, and competent to address their substance use, physical, and mental health conditions.

## Background

As of 2018, over 2.3 million adults comprise jail and prison populations in the United States [1]. While men represent the majority, women are the fastest growing incarcerated cohort, increasing 646% from 1980 to 2010, 50 percent higher than the rate of men [2, 3]. In 2016, nearly 1.2 million women had correctional involvement including probation, parole, and in correctional facilities [4]. Race and ethnicity were important in incarceration; as reported in 2016, for every 100,000 women in the US, 49 Caucasian, 67 Hispanic, and 96 African-American women were incarcerated [4]. As individuals re-entered their communities, many faced challenges navigating complex social services, obtaining Medicaid coverage [5], and accessing substance use disorder (SUD) and primary care treatment [6–9]. Few programs have holistically addressed these concerns. This study describes patient’s experiences of treatment in a specialized medical clinic for women re-entering from incarceration that utilized motivational and trauma-specific approaches.

It has been recommended that women recently released from incarceration participate in evidence-based programming that addresses their higher prevalence of chronic health problems, psychiatric conditions, and SUDs compared with re-entering men [10]. Re-entering women also reported a high prevalence of intimate partner violence (IPV) [11], child abuse [12], and a history of sexual trauma, including from justice system employees [13]. Some relied upon sex work and other high-risk sexual behaviors to cope with SUDs and unemployment [14]. Substance-using re-entering women receiving mental healthcare and wrap-around services, such as childcare, transportation, and employment assistance, have lower rates of access than needed [15]. Craving substances and facing challenges during re-entry can challenge healthy coping strategies, contributing to feeling overwhelmed and relapse [16]. While some research has explored strategies to address these problems, more is needed.

Social support is particularly important for women in re-entry. Mixed method research has shown that social support combined with a feeling of increased self-efficacy moderated the high risk of relapse during re-entry [17]. Conversely, problems in social relationships in the first 3 weeks of re-entry have been associated with increased rates of substance use and hazardous drinking in the following months [18]. Similarly, lack of support and stigma relating to incarceration history and SUDs impeded treatment engagement [19]. Furthermore, a previous qualitative study identified that challenges inherent to re-entry may overwhelm women’s ability to prioritize their health and manage negative emotions, contributing to worsening substance use, mental health symptoms, and recidivism [20]. Even more concerning, re-entry challenges and relapse during re-entry placed individuals at increased risk of both intentional and unintentional overdose [21]. Among individuals with co-occurring disorders and high-risk substance using behaviors, it was recommended to use a harm-reduction approach, work to improve motivation, and strengthen social supports [22]. The high rates of treatment dropout for those with co-occurring disorders highlight the importance of evidence-based strategies to improve engagement in treatment, such as motivational enhancement [23]. An additional challenge is that women with co-occurring disorders also have a relatively high prevalence of medical problems and trauma histories, suggesting potential benefit of a multi-disciplinary and integrated primary medical care approach with support for autonomy, competence, and relatedness [24].

While few primary care models are designed for re-entering women, research has suggested bridging justice systems and health agencies to improve health outcomes [25, 26]. We utilized justice system and other community stakeholders’ input, combined with evidence-based models, to develop a culturally-specific medical care model called Women’s Initiative Supporting Health Transitions Clinic (WISH-TC) [27]. WISH-TC aims to reduce stigma and improve healthcare access via peer-delivered components by formerly incarcerated community health workers (CHWs) using motivational strategies. The program facilitates access to SUD, medical, and mental health treatment to meet patients’ specific needs. Primary care provides an ideal setting for SUD screening, brief intervention, and referral to SUD treatment [28]. Moreover, WISH-TC is trauma-specific according to the trauma, recovery, and empowerment model [29, 30]. Addressing the interconnected “syndemic” of substance use, incarceration, and health risks (including HIV) requires incorporation of proven strategies adapted for this population [31, 32]. Trauma, stigma, and motivation are key leverage points for promising peer community interventions. Given the complexity of the patients and required strategies, it is crucial to understand the voice of the patient as part of the investigation process.

### Trauma-informed and specific care

Trauma-*informed* treatment focuses on avoiding retraumatization through giving clients more control, being supportive, and ensuring physical and emotional safety, including separate spaces for women [33]. Trauma-*specific* treatment, in contrast, addresses clients’ trauma from the remote and recent past through addressing related symptoms and building skills to avoid retraumatization [33]. Research involving justice-involved women with SUDs provided empirical support for addressing trauma and using peer-based, non-stigmatizing, and motivational approaches [34, 35].

### Community health workers and the Transitions Clinic Network model

In 2006, the Transitions Clinic Network (TCN) began employing and training former justice-involved individuals as re-entry CHWs in addition to training physicians to provide healthcare services and increase health literacy for recently released people [36, 37]. These health clinics assist patients in making patient-centered healthcare choices, reduce healthcare costs, offer culturally-specific care from knowledgeable peers, and professionalize careers for formerly incarcerated re-entry CHWs [38]. There is evidence that peer navigation helps decrease relapse rates among patients with SUDs, as well as improve treatment linkage, retention, and patient satisfaction [39]. However, there is limited research addressing medical peer navigation for justice-involved women by re-entry CHWs.

Re-entry CHWs, having experienced and learned to navigate similar struggles, seek to break barriers, engage patients in needed healthcare, and link them to community resources. [41, 41, 42]. Re-entry CHW training includes using motivational strategies to address the multiple needs of re-entering patients. While the re-entry CHW perspective has been reported, research to date has not examined patients’ perceptions of the TCN model [37, 38, 43].

### Self-determination theory (SDT)

SDT is a motivation theory of processes underlying willingness to initiate health behavior change and achieve desired outcomes (see Table 1) [44]. SDT research showed that when providers demonstrate autonomy support (either through training or as a clinical style), patients’ perceived autonomy support mediated their own perceived competence, autonomous motivation, and healthy behaviors [45]. An autonomy-supportive communication style involves taking and acknowledging patients’ perspectives, encouraging and answering questions, supporting initiatives and sense of competence, offering treatment choices, and minimizing control [46]. Validated measures of perceived autonomy support ask about the extent to which the individual feels understood, cared for, accepted, and respected. Autonomy support promotes perceived competence to make healthful behavior changes in areas such as alcohol use, safe sex, tobacco dependence, taking prescribed medications, and weight loss [47–51]. Relatedness was strengthened by staff’s nonjudgmental attitudes and clients feeling their concerns were heard [52]. SDT strategies are appropriate for this population due to the high prevalence of undertreated substance use, medical, and mental health disorders and the motivation required to access treatment. Since women of color are disproportionately imprisoned and experience related social disruption, cultural considerations for SDT are important [53]. As individuals prone to SUDs, stigma, anger, impulsivity, stress, depression, and of varying ethnicity and socio-economic status benefitted from motivation-based interventions, we sought input from our population regarding our specific WISH-TC strategies and relevance of SDT themes in our clinical approach [52, 54].

**Table 1:** Model definitions

Self-determination theory [44]	
Autonomy	The perception of being the origin of one’s own behavior and experiencing volition in action
Competence	The feeling of being effective in producing desired outcomes and exercising one’s capacities
Relatedness	The feeling of being respected, understood, and cared for by others

### Study aims

This exploratory study describes the experiences of women, including those with substance use histories, who participated in a pilot medical clinic for recently released women. We sought to inform our practice and that of others treating similar patients. We conducted a process evaluation of our adapted TCN model, informing how best to meet the women’s practical, medical, and motivational needs. This project is part of a body of work utilizing community based participatory research (CBPR) strategies to investigate our motivational, trauma-specific strategies for helping justice-involved women access the broad spectrum of needed medical care [55].

## Methods

### Clinic description

WISH-TC is based in a primary care clinic embedded in a local academic medical center’s Department of Psychiatry in upstate New York; the clinic treats people with co-occurring mental health diagnoses. The clinic utilizes the national TCN’s culturally-specific model [36, 37]. In accordance with CBPR strategies, we assembled and utilized a community advisory board representing local treatment providers, support agencies, law enforcement, re-entry CHWs, clinic patients, and other stakeholders to solicit basic input regarding the clinic, such as patient enrollment and research strategies [56].

The senior investigator, who has expertise in SDT and trauma-specific interventions, hired three women with histories of SUDs and incarceration to serve as CHWs. The CHWs received weekly training and supervision in SDT-based intervention strategies (process), cultural sensitivity (process), trauma (content), community health (content), and health system navigation (process and content), which they were instructed to use to inform in-person and telephone patient interactions [57]. CHWs receive training in culturally-specific strategies and have various skills, including care coordination to link patients with community resources. CHWs interaction with patients varied from home visits, visits at urgent care or SUD treatment facilities, informal counseling, and frequent supportive phone calls. The intent of WISH-TC was to provide a transition from incarceration informed by these strategies to help address the unique SUD, medical, mental health, and social needs of re-entering women.

### Study recruitment

Patients were primarily told about WISH-TC by CHWs who went to the local jail and women’s prison, supportive housing units, health fairs, SUD treatment programs, as well as by staff in other community programs. A trained college graduate research assistant called patients on the WISH-TC panel and told them about the general study procedures and compensation to recruit them for the study. She interviewed 13 consenting WISH-TC patients as research participants. The recruiter did not previously know the patients and planned to offer interviews to all current or past female re-entering clinic patients on the patient log until content saturation was reached [58]. Eighteen women responded to phone contacts about the interview, four were uninterested in participating, and one did not follow up. Of the 13 participants, 12 planned to return for future appointments; one was no longer a patient. We collected demographic data via a short questionnaire and conducted chart review to determine substance use status. The University of Rochester Research Subjects Review Board approved the study #41229.

### Interview protocol

The trained research assistant conducted semi-structured interviews using a list of open-ended questions with instructions to follow leads as needed to fully answer questions and not ask questions that had already been addressed (see Table 2). The questions asked about helpful and unhelpful experiences regarding CHWs, the physician, clinic strategies and personnel, their broad health concerns, and emotional responses to the clinic. The senior investigator developed the questions in accordance with the SDT process and content of intended clinic strategies. Some of the questions were adapted from the Health Care Climate Questionnaire (α = 0.95), a validated measure of perceived autonomy support that has been shown to mediate perceived competence, autonomous motivation, and healthy behaviors [59]. Interviews lasted between 20 min and 1 h, with a mean of 35 min. Interviews were audio-recorded, transcribed, stored in a locked cabinet, and de-identified prior to analysis. Participants provided written informed consent, and received a $20 gift card and a bus pass or parking validation.

**Table 2:** Interview questions

Category	Sample questions
Community health worker	Do you feel [the CHW] tries to understand how you see your medical situation before suggesting any changes? Do you feel that they care about you as a person? What changes could [the CHW] make to better serve you?
Doctor	How do you feel about the way [the doctor] talks with you about your medical treatment? Do you feel that [the doctor] accepts you whether you follow her suggestions or not? Do you feel you can be open and honest [with the doctor]?
Overall clinic	How easy or difficult was it for you to receive transportation to the clinic? At the clinic did you feel that you were treated in a respectful and in a professional manner? To what extent did the WISH Transitions Clinic meet your needs overall?

### Coding process

The multidisciplinary team coded the de-identified transcribed interviews using a qualitative framework analysis, which identifies a theoretical framework from which to understand the data [60]. The team consisted of two undergraduate students, one researcher-internist, and one CHW. The two providers also participated in the patients’ care. All on the team have engaged in prior framework analysis. The lead author read through all interviews and coded the original transcripts according to the SDT framework. The framework included three categories of autonomy, competence, and relatedness (defined in Table 1). A second investigator reviewed the coding, adding additional codes and marking areas of disagreement. These two investigators came to consensus on all discrepancies, and then met with the other investigators, confirming and resolving any coding disagreements by consensus. Within these three categories, the research team iteratively identified 15 sub-categories delineated in Table 3 [61]. For instance, subcategories of “Supported- Quitting Smoking” and “Supported-sobriety” referred to Autonomy supportive statements regarding these behaviors. They were included as separate categories as the majority of women mentioned such statements. The team recoded the transcripts to reflect the iterative changes with discrepancies reviewed and resolved by consensus [61]. We numerically tabulated quotes in each theme. Some of the quotes overlapped with more than one category or subcategory, so were counted more than once and this will be demonstrated in some of the quotes. Respondent verification validated our analysis to refine explanations and interpretations [62].

**Table 3:** Coding counts

Category	Definition	# of codes	# of women
Autonomy			
Controlled	Perceived lack of agency in decision-making	33	6
Supported	Perceived agency in decision-making	130	13
Supported-quitting smoking	Autonomy supportive statements regarding quitting smoking	16	7
Supported-sobriety	Autonomy supportive statements regarding sobriety	13	7
Competence			
Helped their understanding	Support from clinic staff in understanding health needs	34	12
Health literacy-personal	Women’s existing knowledge of personal health needs	21	4
Health literacy-system	Women’s existing knowledge of navigating health systems	50	8
Set up with needed services	Clinic staff assisting women in navigating health systems	73	13
Staff communication	Staff communication noted as valuable in women’s knowledge of personal health needs	10	8
SUD	Women’s knowledge of their SUD needs	6	5
Relatedness			
Doctor	Support or lack of support from doctor	53	13
CHW	Support or lack of support from CHW	41	13
Other clinic staff	Support or lack of support from other clinic staff	17	12
Overall clinic	Support or lack of support from WISH-TC	22	11
SUD	Support or lack of support from clinic staff regarding SUDs	7	4

## Results

### Demographics

Among the 13 participants, ages ranged from 26 to 61. Eleven of 13 participants reported SUD history. Six were Caucasian, four were African-American, one was Asian, one was American Indian or Alaska Native, and one was Native Hawaiian/Pacific Islander. Twelve were non-Hispanic and one was Hispanic. These percentages reflect county racial and ethnic composition of recently released women during the time period.

We present narrative data according to the categories of SDT (autonomy, competence, and relatedness). Definitions are presented (Table 1) and quotes were shown to distribute relatively evenly across categories. We assigned the women pseudonyms to protect participant confidentiality and to demonstrate utilization of quotes from all participants.

### Autonomy

All women mentioned transportation problems. Attending clinic was a choice they prioritized.

It was winter when I walked up there; it was cold but I had to get up there… it was early in my recovery so I had to get up there. Amy

Women described feeling transformed and improved by making the choice to use the resources and support of the clinic for SUD, physical, support from peer CHWs and/or mental health treatment.

…very positive on how I feel about the changes that I’m making in my life. You know that I’m sober now, that I’m healthy, that I know what’s going on with my body. Stacy

All women noted experiences of autonomy support, most commonly related to quitting smoking, which was mentioned in several interviews.

They’ve encouraged me to try to stop smoking, too… And [the doctor] had gave me all kind of suggestions, but… it’s not a demanding thing… they just flow with me. Pamela

They’re really concerned about my health, my well being, and me being successfully in recovery. And I like that, that gives me strength and courage to do what I need to do because I know I got people that’s backing me. Gloria

Women felt that WISH-TC Staff respected their specific treatment preferences.

When I explain it to them, they’re good without pushing that I try it again, because it didn’t work…they understand that I’m very determined to stay on my maintenance. The one thing that I want to do is to eliminate some of it, so we’re working at doing that. Beverly

I don’t really like taking pills and…I told [the doctor] that and she understood…when I explained what my fears was she suggested physical therapy; I didn’t have to take pills…she listens to what her patients tell her. Deborah

Some women described feeling controlled by clinic staff. Of these women, most mentioned it only a few (i.e., one to five) times. Two individuals comprised the majority of the 33 total codes, with 11 and 15 statements, respectively, of autonomy not being supported. Despite the fact that the CHW went to great lengths providing services to this patient, the patient’s negative perceptions were foremost in her thoughts, perhaps related to difficulties that the patient unrealistically expected the CHW to prevent. Deborah expanded upon an earlier statement that the CHW was trying to act like her mother.

If I say I want to turn right [CHW will] make me go left. And ‘no, this is not the way you want to go…’ She wants me to go where she wants me to go. She doesn’t want me to try nothing I want to do. She wants to control, she’s a control freak. Deborah

Stephanie noted high levels of control and isolation from an IPV relationship at the time that she was struggling with a lack of medical care options. Relatedly, she felt dissatisfied with what she perceived as inadequate care from the doctor and not feeling welcomed at the clinic. Her perceived mistreatment indicated frustration and a lack of understanding of the indication for tests (i.e. infectious disease labs). The lack of lunch-time availability by staff was later addressed in clinic practices.

[I was] misdiagnosed…again not the proper medication, and again not being listened to when I told [the doctor] I had an infection. Is that necessarily all her fault? No. The…infection, yes, that’s completely her fault because I told her over and over again. The office staff, that’s not her fault, not getting the prescription put in the right way, disappearing at lunch… I had to sit there and wait for 25 min. Stephanie

Notably, Deborah made 11 statements and Stephanie made 4 statements describing autonomy support as well indicating their experience was not solely one of feeling controlled. Several women discussed the value of staff communication at the clinic, which facilitated taking charge of their SUD, physical, and mental healthcare.

Everybody on the same page so that’s much easier then like before when I just go to the programs [and]… you have to keep explaining everything to everybody… I can go in there and say ‘I want to talk about my childhood.’ That person already knows what’s going on with me and [is] willing to work with me. Gloria

### Competence

Patient intake in jail, prison, transitional houses, and other places women were in the community helped them feel competent to get their health needs met, avoiding the frustration and futility of finding providers on their own.

If it weren’t for them coming in and presenting the program to me in the jail, I may have gotten frustrated trying to find a doctor on my own. I may not have gone to a doctor on my own. Cynthia

Upon re-entry, the clinic supported women’s competence to get healthcare before they became overwhelmed by other concerns through flexible appointment scheduling.

They met all my needs quickly. And getting out of prison is… mind blowing… I didn’t know where I was gonna get medical help and I needed it bad. Beverly

CHWs’ knowledge of community resources and advocacy for their patients helped all of the women set themselves up with necessary services like SUD, housing, and medical specialists.

When I relapsed, I called [the CHW] all the time while I was using to help me get in somewhere, help me find a place to stay, a safe place while I was waiting for a bed at [inpatient SUD treatment]. Amy

Most women felt that the clinic and its staff helped their understanding of their health situation through caring and using language they understood.

When [the doctor] explained the tests and what they were and why they were helpful for me just on a personal level. It felt like she actually cared about what I was going through to offer things that I wasn’t asking for. She expanded on my needs. Jane

I was very frustrated trying to get into see this one surgeon and [CHW] took extra time to make sure I understood that she was gonna work to get my medical records and to get me in as soon as she could. Beverly

Several women raised health literacy-related issues. Only a few showed an absence of health literacy, while most combined a lack and presence of health literacy or solely presence of health literacy. Some women indicated competence regarding their health combined with autonomy in making the choice to take action.

I had…a boil on my butt and I was kind of embarrassed to tell her, but I couldn’t take it no more ‘cause it was painful… I said I need antibiotics. I didn’t want to tell for what and then she asked me …And I didn’t feel that bad after that because like she immediately knew what to do and did it and it went away. And she asked me about it today: ‘I know that had to be painful’ so. I felt good that she said that ‘cause she knew that I was going through pain. Stacy

All women mentioned ways that the clinic helped them understand their needs and act accordingly, demonstrating dual competence and autonomy, including one woman who said it helped her use self-care rather than emergency care.

She had me coming [to the clinic] once a week to keep me from going to emergency. So far I’ve been doing great. I haven’t [had] any problems to where I had to go to emergency because…she had me coming in like that. If there is something going on with me and I can’t get a hold of the doctor…I’m gonna go to emergency. That’s what I do. Sarah

### Relatedness

Most women expressed gratitude for CHWs who helped set up medical appointments before leaving jail and prison and kept them on track for SUD, physical, and mental healthcare. This support helped women feel cared for and understood by clinic staff.

I just generally was really grateful for the [clinic] program because… when I got out, I already had a scheduled appointment for the doctor and I hadn’t been in years and I had had a lot of concerns about my health…when I got out of jail… I didn’t end up just like giving up… I was already set up for when I stepped out one door to go into the next door… there were other times I was in jail, I had all these thoughts in my mind about getting sober and getting clean and doing all the right things. But as soon as I stepped out the door, I took a left instead of a right; I didn’t pursue finding a doctor; I didn’t pursue getting clean… I think probably part of it was ‘cause I didn’t have those things set up for me before I got out. Cynthia

Women showed emotional self-awareness. One woman described how the doctor supported her through her extreme anxiety, utilizing medication rather than drugs or alcohol, and the changes she noticed in herself.

… When I was really, really anxious [the] first time [at the clinic], [the doctor] acknowledged my anxiety … She just allowed me to feel the anxiety…With the anxiety medicine I’m able to actually speak instead of hiding. That’s helped immensely, but she was able to diagnose that and find something that does help…I’m not having panic attacks like I used to. I’m able to actually sit and talk to somebody with a little bit of nervousness, but not as bad as it used to be. Joyce

All women described experiences of relatedness support with their doctor and CHW.

… When you first enroll with a new doctor… you’re [not] always comfortable right away, and that’s how it was [at] the beginning of her being my doctor. But [then]… I saw her potential and how eager she is and whatever is going on with me medically, she always does her best to address it. So it makes me feel comfortable that I can confide in her because it’s for my best interest… whatever she does. Gloria

I think that when [CHW] came into the jail her actually presenting that [transitions clinic] program in itself was a big one for me. I didn’t have to come out of jail and try to figure out what doctor to go to. And the fact that they’re following up with how I’m doing since I got out and how I feel about the doctors and where they referred me to. Cynthia

However, a few women recounted feeling that a CHW thought she was superior to them and others felt uncomfortable with the suggested phone calls which were meant to address CHW difficulties reaching patients who often lost phones or changed numbers and to help them take initiative in their healthcare.

I don’t think [the CHW] respected me very much and it was sad because she’d been in jail too. But because she… got a job… I felt like she felt ‘I’m better than them.’ Some people get that… grandiosity air with them. Deborah

She’s insisting that I call her at least weekly, which I’m very bad at. I’m gonna try and give it my best effort. I’m very forgetful at things like that and I’m not very much of a phone person, so I kind of shy away from that. I said ‘well you haven’t heard from me, so everything must be going well’… just the urgent need of constant contact that’s like scary, foreign you know. Joyce

Most women experienced relatedness support from other clinic staff. For example, the office staff acquired knowledge of SUDs and applied it in patient interactions.

They show that they care. I missed two appointments and…the secretary asked me ‘can we make another appointment?’… And I told her… ‘I can’t, I’ve been using, I’m going into [inpatient SUD treatment].’ And she goes, ‘Honey, whenever you’re ready you just call and we’ll set that up for you.’ She didn’t make any kind of comment like ‘you’ve missed two appointments… you’re gonna have to pay a $25 fee… when I called she remembered, I said, ‘I’m ready to make an appointment. She goes ‘I can’t wait for you to come in’ and that was great. Amy

### Process evaluation

The above data resulted in clinic procedural changes. The staff was trained to fine-tune communication strategies regarding emotional dysregulation and potential boundary concerns from trauma, stigma, and racism, such as when patients are intoxicated, late, or show up unexpectedly. They also developed systems to provide information or help with prescriptions, finding community laboratories, transportation, or follow-up imaging or referral appointments. CHWs were trained to respond to patient cues regarding the amount of contact, maintaining appropriate boundaries, maximize patient control, and avoid overwhelming them. In addition, an orientation and hand-outs were added to educate new patients about the roles of the physician and CHWs to maximize systemic health literacy. Lastly, the clinic fine-tuned processes to ensure that the staff is available if needed during lunchtime.

## Discussion

These medically and socially complex women generally viewed the specialized clinic strategies positively, which has implications for others caring for similar patients. Previous studies have shown that SDT strategies are effective in addressing a variety of health risk behaviors, and we found that this model was appropriate for use with women in re-entry [47–51]. Regarding autonomy, staff presenting WISH-TC to women while incarcerated helped empower them to make the decision to seek healthcare upon release and get their needs met quickly, despite system barriers. Women noted how autonomy support from various clinic staff helped them to develop motivation and navigation skills and work towards personal goals such as quitting smoking and maintaining sobriety. Women prioritized coming to the clinic, despite system challenges, as they saw the services as essential for their recovery. Women developed competence in terms of building upon their existing health literacy, learning about community resources through CHWs, and gaining a better understanding of their personal health needs through interactions with the physician and other clinic staff. Program staff reached out to contact participants prior to release from incarceration as well as in community locations to provide assistance in scheduling clinic appointments, which facilitated women’s navigation competence. Relatedness contributed significantly to women’s satisfaction with WISH-TC. All noted experiences of relatedness support with their physician and CHW that made them feel comfortable and understood at the clinic. Staff were viewed as nonjudgmental and supportive to women entering SUD treatment. Women noted that staff’s knowledge of their personal history made them feel comfortable discussing trauma and communicating openly about their concerns. The sum of this feedback supports the utility of the program and gives indications of what is helpful and what is not.

Women indicated that staff engaged them, built upon their health literacy, and developed supportive relationships. While only CHWs and physicians are trained in the Transitions Clinic model, our data helped staff of various roles, including nurses and reception staff acquire strategies to improve their impact on the women’s experiences at WISH-TC, including being trauma-informed. Setbacks, including substance use are likely during re-entry and continued nonjudgmental support as displayed by clinic staff is important for successful recovery [17]. Satisfaction encompassed much more than their experience with medical providers, although the physician was cited as a reason why many felt comfortable and willing to return. This study highlights the importance of sensitivity and addressing stigma in building trust among women with SUD and trauma histories, difficulty with emotional regulation, and minimal resources. Many described attitudinal and behavioral transformations. Personal and systemic health literacy was an issue for many, with the majority of women showing a combined lack and presence of health literacy. Building upon existing knowledge, rather than attempting to teach from the ground up, can be effective in improving health literacy [36, 51].

While two women described autonomy control from clinic staff, it is surprising that more did not feel this way. All women experienced high levels of control related to incarceration, though only 7 of 13 mentioned additional external control such as IPV relationships or other assaults that limited their freedom and often resulted in judgment by community and family. They also experienced community supervision requirements by probation or parole. One patient in this cohort but not in this interview study described a sexual assault from a probation officer. Rates of prior physical or sexual abuse among incarcerated girls are 80–90% [63]. Social service rules can result in being denied services if women are late without inquiries as to the reasons, hence not using trauma-informed strategies. Notably, the two women in this study with the most negative comments were experiencing even more external control than other participants, including IPV. It is possible that this external control generalized to their feelings about the physician and CHWs, but is informative regarding a need to be sensitive to boundaries and dysregulation. Previous research has shown that autonomy support from physicians is predictive of autonomous motivation to quit smoking [51]. Similarly, although women reported autonomy supportive strategies regarding smoking cessation, some were not ready to quit. Future studies could investigate the role of feeling controlled in multiple arenas for women who did not quit smoking. As noted above, we incorporated even more sensitivity to feeling controlled into already autonomy-supportive practices.

Difficulty regulating emotions and setting boundaries could relate to prior trauma for both the patients and the peer CHWs in their interactions and may have negatively shaped some women’s perception of the peer CHWs. A few mentioned feeling that the CHWs thought they were superior, described distrust, and felt they were contacted too often. Emotional dysregulation in those with trauma histories can increase sensitivity to perceived control, and peers were trained to avoid escalation [33]. Additionally, one woman expressed resentment that the CHW would not write a medical order, which the CHW could not do. This displayed a need for more patient education on the roles and responsibilities of clinic staff, which we included in our subsequent patient orientation. As the clinic operates in a medium-sized city and CHWs are peers, it is unsurprising that boundary issues arose in some women. Future studies could investigate these issues more specifically, including the role of boundaries in peer-patient interactions.

Race concordance is not required to build trust between patients and health providers, but it can be helpful [64, 65]. Perceived personal similarity also helps build trust between physicians and patients [65]. However, our population is unique and worthy of further study, as they are re-entering women accessing health services from peer CHWs as well as physicians [64, 65]. SDT-based strategies avoid controlling statements, which helps to build trust with patients [44, 45]. CHWs in this clinic provide care using an SDT-based intervention. Further study could examine race concordance in SDT-based interventions with CHWs. Although we made changes related to participant feedback, sensitivity regarding emotional dysregulation and stigma would be important for other institutions serving women with trauma histories and those re-entering from incarceration.

### Limitations and strengths

The semi-structured interview may not have captured an exhaustive list of ways the clinic helped or hindered the women. However, the interviews were conducted by a research assistant who did not work in the clinic and participants did feel comfortable expressing negative views. Except for one participant, this study only included those who plan to return. Additionally, this clinic is based in a Department of Psychiatry and is set up to include people with co-occurring psychiatric diagnoses, so it could be a select population on that basis. The prevalence of SUD among re-entering women is approximately 70% [66] and 90% in our population [67]. Simultaneously, the prevalence of depression among re-entering women is described as 56% generally and 88% in our population, but women were not recruited to the clinic on the basis of SUD or depression [68]. Childcare or parenting issues may be underrepresented as interview questions focused on the clinic [69]. Strengths of this exploratory study include the multidisciplinary team, including a formerly incarcerated person, and respondent verification. Study limitations should be viewed in the context of an understudied population giving feedback on a novel program.

Future studies could examine in greater detail the understudied area of health literacy, both qualitative and quantitatively. For example, what types of health literacy are present that providers can build upon, and what types are absent in this population? Also, future research could consider the impact of stigma, and peer-support with the use of CHWs in healthcare settings and its impact on perceived care. Since patients are not at the same stage in their recovery as the peer CHWs, they can feel resentful and insecure and this issue is worthy of future study. It would be helpful to randomize re-entering women to a program such as ours or standard treatment to compare perceptions of the two programs.

## Conclusion

Re-entering women face challenges to accessing quality medical care that meets their needs. WISH-TC’s integrative design and staff’s understanding of the unique needs of this population have helped women feel supported, motivated, and competent to address their substance use, physical, and mental health needs. It is worth further exploring ways to improve health literacy in this population, as well as dynamics between peer CHWs and patients. While this study informed our clinic practices, we believe it can also inform others working clinically and using research to meet complex needs of justice-involved women, especially other programs using a peer-to-peer model with re-entry CHWs. Our study models for other agencies serving justice involved women how integral nonjudgmental staff, trauma-informed care, and minimizing systemic barriers to access care are to women’s engagement in treatment.

## Abbreviations

CBPR: community-based participatory research
CHWs: community health workers
IPV: intimate partner violence
SDT: self-determination theory
SUD: substance use disorder
PTSD: post traumatic stress disorder
TCN: Transitions Clinic Network
WISH-TC: Women’s Initiative Supporting Health Transitions Clinic
